# Influence of walking on knee ligament response in car-to-pedestrian collisions

**DOI:** 10.3389/fbioe.2023.1141390

**Published:** 2023-04-13

**Authors:** Wentao Chen, Jisi Tang, Wenxuan Shen, Qing Zhou

**Affiliations:** State Key Laboratory of Automotive Safety and Energy, School of Vehicle and Mobility, Tsinghua University, Beijing, China

**Keywords:** car-to-pedestrian collisions, knee ligaments, ligament properties, walking effects, material uncertainty

## Abstract

Pedestrians are likely to experience walking before accidents. The walking process imposes cyclic loading on knee ligaments and increases knee joint temperature. Both cyclic loading and temperature affect the material properties of ligaments, which further influence the risk of ligament injury. However, the effect of such walking-induced material property changes on pedestrian ligament response has not been considered. Therefore, in this study, we investigated the influence of walking on ligament response in car-to-pedestrian collisions. Using Total Human Model for Safety (THUMS) model, knee ligament responses (i.e., cross-sectional force and local strain) were evaluated under several crash scenarios (i.e., two impact speeds, two knee contact heights, and three pedestrian postures). In worst case scenarios, walking-induced changes in ligament material properties led to a 10% difference in maximum local strain and a 6% difference in maximum cross-sectional force. Further considering the material uncertainty caused by experimental dispersion, the ligament material property changes due to walking resulted in a 28% difference in maximum local strain and a 26% difference in maximum cross-sectional force. This study demonstrates the importance of accounting for walking-induced material property changes for the reliability of safety assessments and injury analysis.

## 1 Introduction

Lower extremity injuries are common in car-to-pedestrian collisions (Chidester and Isenberg, 2001; Yang, 2005; Mallory et al., 2022). The injury patterns include bone fractures and knee ligament ruptures, which depend on loading conditions (e.g., impact position and impact velocity) and pedestrian states (e.g., posture, height, and orientation). Ligament injuries primarily occur in the four main ligaments of the knee: anterior cruciate ligament (ACL), posterior cruciate ligament (PCL), medial collateral ligament (MCL), and lateral collateral ligament (LCL) (Klinich and Schneider, 2003; Kerrigan et al., 2012; Mallory et al., 2022). Although ligament injuries are not life-threatening, they seriously affect the quality of life of patients and cause a heavy financial burden. In recent years, autonomous emergency braking (AEB) systems have become increasingly popular. When encountering pedestrians, AEBs can reduce impact speed and induce vehicle pitching to reduce bumper height, thereby affecting the risk of ligament injuries (Matsui, 2005; Mo et al., 2013; Asgari and Keyvanian, 2019).

**FIGURE 1:**
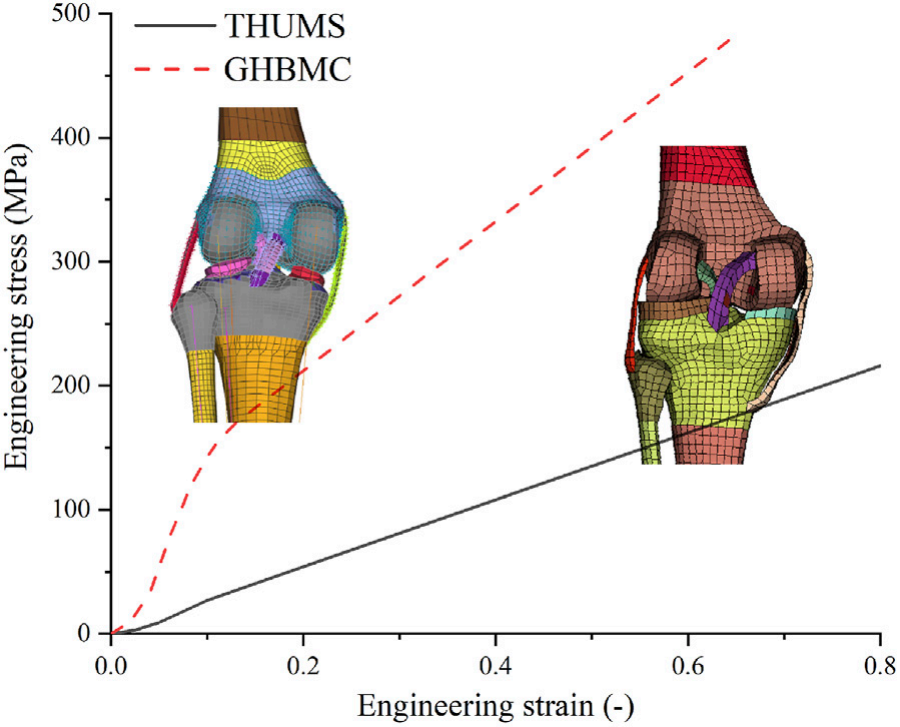
Material curves of the knee ligaments of THUMS and GHBMC models. THUMS, Total Human Model for Safety; GHBMC, Global Human Body Models Consortium.

Numerical simulation is essential in pedestrian injury analysis. It can integrate the learnings obtained by individual methods (e.g., animal and volunteer tests) and provide considerable insight into complex loading conditions (e.g., strain distribution and injury evolution). Pedestrian impact simulations mainly use human body models (HBMs), such as Total Human Model for Safety (THUMS) and Global Human Body Models Consortium (GHBMC), to provide tissue-level responses (e.g., deformation and forces of knee ligaments in pedestrians) that are important in injury analysis. Despite THUMS and GHBMC models having geometric differences due to individual variability, the geometry of the knee joint has a relatively high biofidelity (Kazemi et al., 2013), while the material data of the knee ligaments are less reliable. THUMS and GHBMC use significantly different material curves for their respective knee ligaments (Figure 1). In addition, although both models have been validated on whole-body responses (Wu et al., 2017; Panday et al., 2021), the reliability of the knee ligament responses has not been carefully addressed. For example, the knee joint of THUMS is said to be validated based on dynamic 4-point bending and lateral loading experiments (Toyota Central R&D Labs Inc, 2018). However, the simulation results of the 4-point bending case using the knee joint of THUMS showed that the kinematic response did not match well with the experiments (Figure 6), challenging the plausibility of the dynamic response.

The influences of pedestrian posture (Tang et al., 2016; Tang et al., 2020), knee-bumper contact height (Mo et al., 2013; Mo et al., 2014), bumper stiffness (Nagasaka et al., 2003), etc., on ligament response have been studied in pedestrian injury analysis. However, the influence of walking-induced material changes on ligament response has not been well assessed (Shrinivas et al., 2023). Ligaments are biomaterials whose material properties and responses are sensitive to working conditions. Pedestrians may experience a walking process before traffic accidents. The walking process applies cyclic loading on knee ligaments and increases the body temperature by accelerating metabolism. Both cyclic loading (Sekiguchi et al., 1998; Zitnay et al., 2020; Firminger and Edwards, 2021) and temperature (Dorlot et al., 1980; KarisAllen and Veres, 2020; Chen and Zhou, 2022) influence the material properties of ligaments. However, it is not clear to what extent walking-induced material changes affect the ligament response of pedestrians in car-to-pedestrian accidents, and whether they are worth considering. To answer these questions, in this study, we first recalibrated the knee joint model of THUMS. Then, using the modified model as the baseline, we analyzed the effect of walking-induced material changes on pedestrians’ ligament responses under several accident scenarios.

## 2 Materials and methods

### 2.1 Recalibration of knee ligament material

**FIGURE 2:**
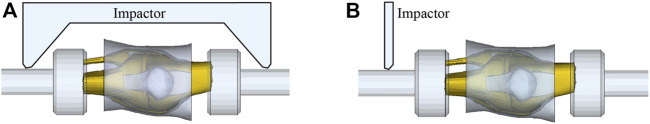
Models for recalibration of the knee ligament material: **(A)** Four-point bending; **(B)** Three-point bending.

In this study, we adopted the THUMS pedestrian model V4.02 (LS-DYNA platform) and referred to it as the “original model.” As aforementioned, the reliability of the ligament material data of the knee joint has not been well considered. Therefore, we first modified the knee ligament material and recalibrated the knee joint model of THUMS. The THUMS knee joint model has been validated against the 4-point bending and lateral loading experiments (Kajzer et al., 1997; Kajzer et al., 1999; Bose et al., 2004; Bose et al., 2008). However, the lateral loading experiments are not representative of pedestrian injuries in real-world crashes (Yoganandan et al., 2012). Hence, instead of the lateral loading tests, we used the 3-point bending tests for validation (Bose et al., 2004; Bose et al., 2008). The 4-point bending model provided by THUMS was used for our recalibration (Figure 2A). By replacing the impactor, we obtained the 3-point bending model (Figure 2B).

THUMS uses the *MAT_SIMPLIFIED_RUBBER (i.e., MAT181) material card in LS-DYNA to characterize the knee ligament material. This material card was retained because it was able to meet the needs of this study. The principle Kirchhoff stress is given by:

$$\tau_{ii}=f\left(\lambda_{i}\right)+K\left(J-1\right)-\frac{1}{3}\sum_{k=1}^{3}f\left(\lambda_{k}\right)$$ (1)

where *f* is the uniaxial tensile curve (or curves at different strain rates), *K* is the bulk modulus, and *J* is the relative volume change. The material card can directly accept experimentally measured material curve *f* without parameter fitting (Bois, 2003). Hence, we next described mainly the material curves used.

**FIGURE 3:**
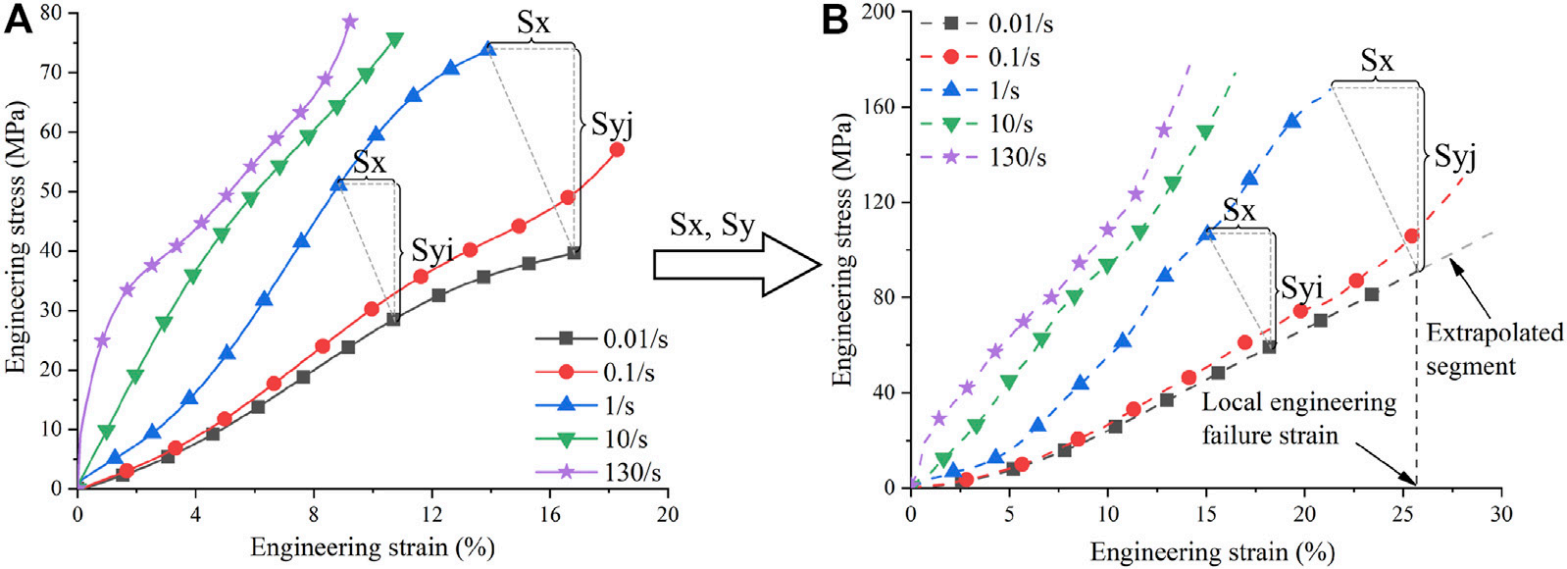
Strain rate scaling of ligament material: **(A)** Tensile curves of porcine LCL at different strain rates (Bonner et al., 2015); **(B)** Tensile curves of human MCL after strain rate scaling. Sx is the scaling factor of strain, Syi is the scaling factor of stress at the *i*th data point, and Syj is the scaling factor of stress at the *j*th data point. MCL, Medial Collateral Ligament; LCL, Lateral Collateral Ligament.

The literature has much of the material property data of human knee ligaments, but there has not been any single study that provides the material properties of all four main ligaments that could be used in simulations. Therefore, we had to assign stress-strain curves to ligaments by combining the results of different papers. In the original model, the four knee ligaments were given the same material curve. However, experiments have found that MCL is significantly stiffer than the other three knee ligaments, and there is no evidence that the other three ligaments have significantly different mechanical properties (Buschmann and Bürgisser, 2017; Smeets et al., 2017; Ristaniemi et al., 2018). Hence, we assigned the experimental curves of MCL and LCL measured by Smeets et al. (2017) to MCL and LCL, respectively, and the material curve of LCL was also given to ACL and PCL. The MCL material curve measured by Smeets et al. (2017) is very close to that measured by Quapp and Weiss (1998). So, we consider these material data to be reliable. When simulation needs material data beyond the strain level of the input curve, LS-DYNA will automatically extrapolate the input data. To ensure the material data used by simulations is under control, the ligament curves of the original THUMS model are smoothed and extrapolated to a large enough strain level, eliminating the yield segment (Figure 1). The similar treatment was adopted to the experimental curves before entering into the material card (Figure 3B).

Thereafter, we adjusted the bulk modulus of the material card. *MAT181 uses bulk modulus, rather than Poisson’s ratio, to control the volume variation. Although the Poisson’s ratio of ligaments is controversial (Lynch et al., 2003; Vergaria et al., 2011; Swedberg et al., 2014; Gatt et al., 2015), most constitutive models assume that the ligament material is incompressible (Galbusera et al., 2014). In theory, incompressible materials have an infinite bulk modulus, while if the bulk modulus is too large, it may cause numerical instability for the critical time step will be too small. After trial and error, we increased the bulk modulus of the four ligaments to 5E5 MPa.

The failure strains of each ligament were collected from the literature (Quapp and Weiss, 1998; Kerrigan et al., 2003; Van Dommelen et al., 2005a; Chandrashekar et al., 2006; Wilson et al., 2012; Smeets et al., 2017). These studies have similar experimental conditions to those of Smeets et al. (2017), i.e., quasi-static experiments at room temperature without cyclic loading. Experimentally measured global engineering failure strains were converted to local true failure strains using the method of Mo et al. (2012). The local true failure strains were then input to *MAT_ADD_EROSION for failure characterization (Table 1). In the original bending cases of the THUMS model, the kinematic response did not match the experimental results. Hence, the velocity boundary conditions were adjusted for 3-point and 4-point bending separately, until the kinematic response (time vs. knee bending angle) of simulations matched that of the experiments. The ligament material cards were then set as aforementioned, and the dynamic responses (knee joint moment vs. knee bending angle) are shown in Figure 6.

**TABLE 1:** Failure strain of the four knee ligaments.

	ACL	PCL	MCL	LCL
Global engineering failure strain (%)	28.0	28.0	24.0	24.0
Local true failure strain (%)	24.7	19.3	24.5	22.9
Local engineering failure strain (%)	28.0	21.3	27.8	25.7

### 2.2 Ligament material properties accounting for strain rate effect

The strain rate of collateral ligaments at a 40 km/h pedestrian impact is about 30–50/s (Van Dommelen et al., 2005b). Accounting for strain rate effect improves material accuracy, making ligament behavior more biofidelic under dynamic collisions. Although it is not difficult to account for the strain rate effect in *MAT181, by a table consisting of material curves corresponding to different strain rates, the original THUMS model does not consider the strain rate effect for knee ligaments.

The effect of strain rate on ligament material was taken from Bonner et al. (2015), who measured porcine LCL material at five different engineering strain rates (0.01, 0.1, 1, 10, and 130/s) (Figure 3A). Heterogeneity exists between data from different studies owing to different experimental subjects or settings. The heterogeneity makes it difficult to directly use the experimental curves of pigs in pedestrian simulations. Therefore, we abstracted the “relationship” of the experimental curves at different strain rates, and then applied this “relationship” to human baseline material curves to obtain material curves that can be used in pedestrian simulations. The recalibrated ligament material curves were measured at 0.02/s, close to 0.01/s. Hence, using the data from Bonner et al. (2015), we calculated the scaling factors of the material curves at other strain rates (i.e., 0.1, 1, 10, and 130/s) relative to the material curve at 0.01/s. We assumed that the strain rate effect for the four knee ligaments was the same. The scaling factors were multiplied by the recalibrated ligament material curves to obtain material curves at different strain rates that could be used in pedestrian simulations (Figure 3).

The scaling factors were calculated as follows. The number of data points of each curve in Figure 3A was adjusted to the same by linear interpolation (i.e., 100), where the strain values were generated in equal increments. Because the strain values were equal difference series, only one scaling factor was needed for strain. The division of the endpoints (i.e., failure strain) at different strain rates by the endpoint at 0.01/s was the scaling factor for strain (horizontal axis). In contrast, the differences in the stress values were not uniform, so the scaling factors of stress needed to be generated for each data pair. The *i*th scaling factor was the stress of the *i*th data point at a higher strain rate divided by the stress of the *i*th data point at 0.01/s. The number of data points of the effective curve segment (before the failure strain) of the baseline material curves was also adjusted to 100, and the strain values had an equal increment. Because we used the engineering stress-engineering strain curves in *MAT181, the local true failure strains were further transformed into local engineering failure strains to determine the effective curve segments for each ligament (Figure 3B). Then, the effective curve segment was multiplied by the strain scaling factor and stress scaling sequence, respectively, to produce its material curves at different strain rates (Figure 3B). Note that although the recalibrated material curves of LCL, ACL, and PCL were the same. The resulting strain rate curve series differed due to their different failure strains. All curves after strain rate scaling were smoothed and extrapolated, eliminating the yield segment. These curve series were then input into the material card to characterize the strain rate effect for ligaments.

There is another issue that needs to be addressed to account for the strain rate effect of ligaments in simulations. Previous studies have found that the global strain of ligaments differs greatly from the local strain due to the uneven deformation of ligaments in tensile tests (Woo et al., 1983; Kawada et al., 1999; Slane et al., 2017). The same is true for strain rate, and the difference is even greater (Zitnay and Weiss, 2018; Rocas et al., 2021). For example, the material curves measured at 0.02/s global strain rate should have a local strain rate larger than 0.02/s. And this effect has been reflected in the bending simulations. When the material curve series obtained by the above operations were used in bending simulations, the simulation results were much stiffer than the experimental results.

We examined the strain rates of the ligament elements of the 3-point and 4-point bending simulations and found that most ligament elements had a local strain rate fluctuating around 10/s. Consequently, we viewed 10/s as the equivalent local strain rate for our baseline material curves measured at a global strain rate of 0.02/s. Because of the lack of effective local strain rate measurements for ligaments at this stage, it was assumed that the scaling factors of local strain rates were the same as those of global strain rates, such that the scaling factors of the material curve at global *y*/s relative to that at global *x*/s were the same as those of the material curve at local *y*/s relative to that at local *x*/s. The strain rate scaling was achieved by calculating the scaling factors of the material curves at 0.01, 0.1, 1, and 130/s relative to the material curve at 10/s. Such scaled curves enable the simulation results of the 3-point and 4-point bending cases differed by less than 1% from those in Figure 6, having a good agreement with the experimental results.

### 2.3 Influence of walking on ligament material properties

Walking affects ligament material properties by applying cyclic loading and increasing body temperature, and the two factors are coupled to each other (Chen and Zhou, 2022). Under some cyclic loading conditions, ligament stiffness increases with increasing temperature, while under other cyclic loading conditions, ligament stiffness is weakened by increasing temperature. Therefore, we considered their combined effect in this study.

**FIGURE 4:**
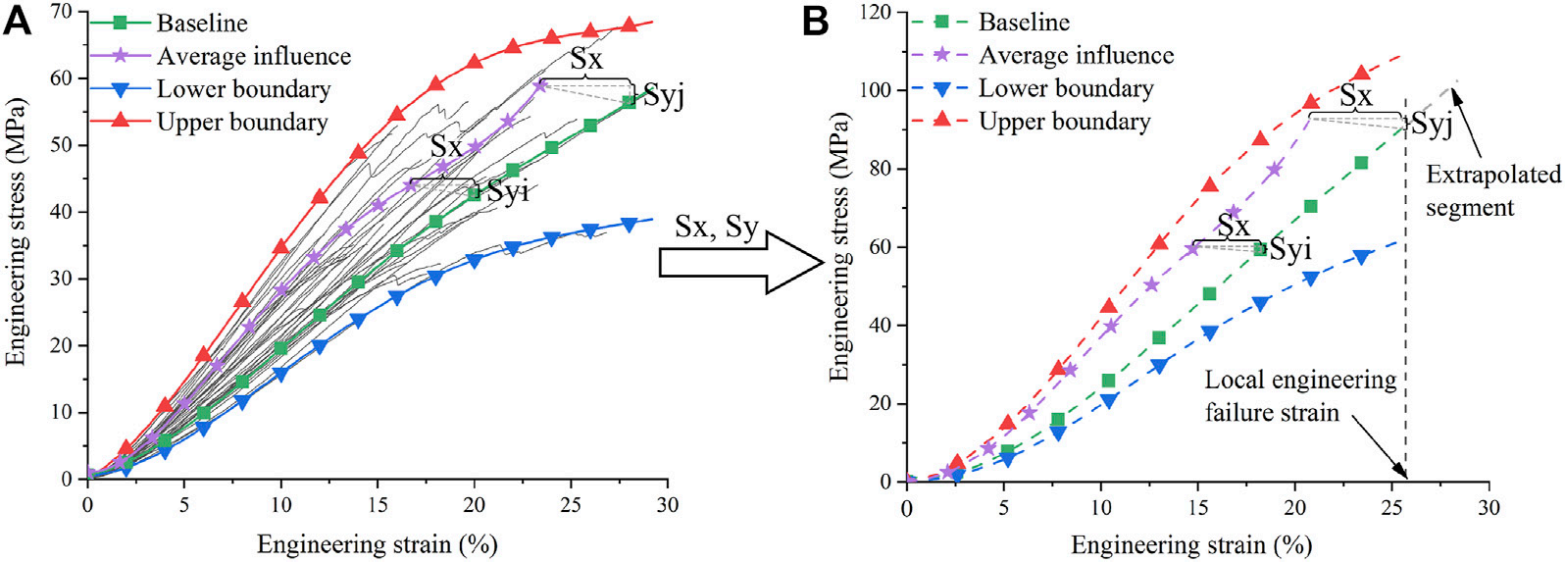
Walking scaling of ligament material: **(A)** Tensile curves of porcine MCL under different walking conditions (Chen and Zhou, 2022); **(B)** Tensile curves of human MCL after walking scaling.

Influence of walking on ligament material was extracted from our previous paper using porcine MCL as experimental subjects (Chen and Zhou, 2022). We calculated the scaling factors using the average material curves before and after walking (i.e., average scaling method). Specifically, we used the “baseline” curve measured at the experimental condition close to Smeets et al. (2017) (i.e., at room temperature without cyclic loading) and the “average influence” curve measured after a short-term walking process (Figure 4A). The scaling factors of the “average influence” curve relative to the “baseline” curve were calculated. It is very common to use average experimental curves for simulation, which requires a single specific input to describe each quantity. However, experiments inevitably have variations, especially in biological experiments. For example, the elastic modulus of MCL measured by Smeets et al. (2017) has a mean value of 441.8 MPa and a standard deviation of 117.2 MPa. The standard deviation is nearly 1/3 of the mean value; thus, there is a large uncertainty in the material parameters. Deterministic simulation result using the average material curve can hardly representative of the worst case. However, the worst case analysis is important in injury analysis. Hence, considering the material uncertainty, we also used the upper and lower boundaries of the experimental curves in simulations, calculating the scaling factors of the upper and lower boundaries of the experimental curve envelope with respect to the baseline curve. The calculation method was similar to strain rate scaling.

Walking affects the ligament material much more slowly than the crash duration, so it was considered to change mainly the baseline curve before the collision. We assumed that strain rate and walking affect ligament materials independently, so that the two factors can be scaled separately. The effective curve segment of the baseline material curves was multiplied by the scaling factors, producing the curves influenced by walking (Figure 4B). The generated material curves corresponded to the global quasi-static state (i.e., local 10/s), which were further multiplied by the scaling factors of strain rate to obtain curves at different strain rates. All curves after scaling were also smoothed and extrapolated, eliminating the yield segment, and entered into the material card.

Before simulating the effect of walking on pedestrian ligament responses in car-to-pedestrian collisions, we tested the effect of walking-induced material changes on 3-point and 4-point bending. The maximum local true strain (hereafter referred to as maximum local strain) and maximum cross-sectional force of MCL were compared. If ligament failure was set in simulations, the maximum local strain of the cases with MCL failed is approximately equal to the failure strain, making it difficult for the maximum local strain to reflect the relative injury risk between these cases. To obtain comparable maximum local strain, ligament failure was not included in simulations when analyzing the effects of walking on ligament response. Ligament failure was only included in the 3-point and 4-point bending models for recalibration purposes.

### 2.4 Simulation matrix of car-to-pedestrian collisions

**FIGURE 5:**
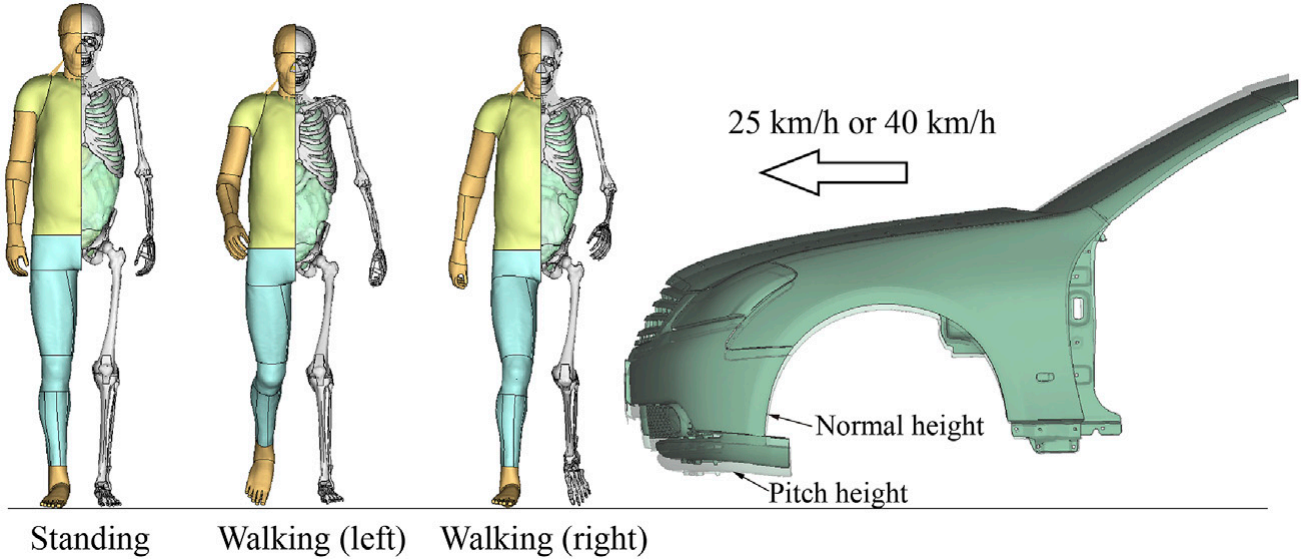
Illustration of pedestrian accident scenario.

The ligament material properties obtained in the above process were used in car-to-pedestrian collision simulations to evaluate the influence of walking on pedestrian ligament response. The simulation model was the same as Tang et al. (2016), and the car model has been validated (Tang et al., 2016). As shown in Figure 5, only the frontal structure (A-pillar and forward) was retained in the vehicle model, and the mass properties of the entire vehicle were compensated by using an attached mass at the center-of-mass position. The pedestrian was laterally oriented relative to the vehicle, along the centerline of the vehicle. Twelve different crash scenarios were simulated (Figure 5).

The pedestrian had three different postures: standing, mid-stance gait in walking with left and right legs forward, respectively, the same as those set by Tang et al. (2016). When the pedestrian was standing on the ground, the bumper impacted the middle of the knee joint. This position was referred to as the “normal” height. Execution of AEBs before a collision can reduce bumper height due to pitching. We simulated the emergency braking process of a typical sedan on Carsim platform and found a maximum pitch angle of 1.7°. The 1.7° pitching resulted in a downward shift in the bumper height of approximately 75 mm. This impact height was referred to as the “pitch” height. The current pedestrian crash regulations mainly test at 40 km/h, and AEBs can generally reduce a 40 km/h impact to about 25 km/h (Gruber et al., 2019; Leo et al., 2020). Therefore, we simulated 25 km/h and 40 km/h impact speeds. Altogether, the simulation matrix included three pedestrian postures, two impact heights, and two impact speeds.

The influence of walking (lower vs. upper boundary curves) was tested in the full simulation matrix (12 cases). The case most affected by walking was then singled out and re-simulated using the average scaling method (baseline vs. average influence curves) to estimate the discrepancy owing to material uncertainty. Maximum local strains and maximum cross-sectional forces of knee ligaments were reported to evaluate the difference in ligament response. The strain rate effect was included in all the simulations. Also, we did not include ligament failure in pedestrian simulations.

## 3 Results

### 3.1 Comparison of simulation results of the new and original materials

**FIGURE 6:**
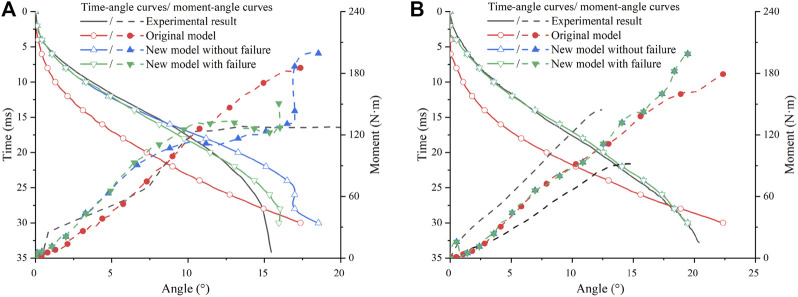
Comparison of simulation results for bending cases: **(A)** Three-point bending; **(B)** Four-point bending. The experimental corridor is from Ivarsson et al. (2004).

Both ligament failure on and off in the material model were simulated (Figure 6). In the 4-point bending case, the curves with and without failure had no difference since no ligament failed in the simulation. ACL failed in the 3-point bending case, which was observed in the experiments. The newly recalibrated material model correlates better with the experiments than the original material model, so the recalibrated material parameter setting can be considered reasonable.

**FIGURE 7:**
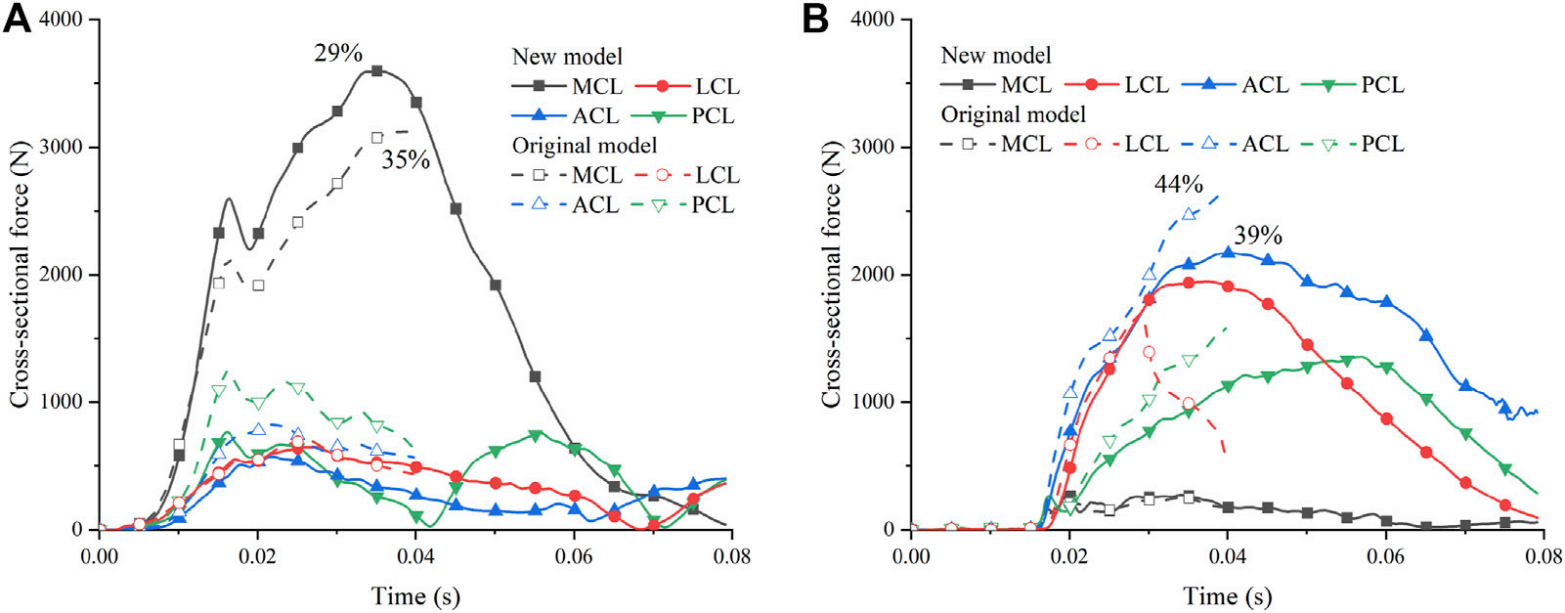
Comparison of simulation results for the pedestrian accident: **(A)** Ipsilateral leg; **(B)** Contralateral leg. The percentage numbers indicate the maximum local strain.

The recalibrated material setting was then applied to the pedestrian model. We compared the simulation results of the new and original THUMS models in car-to-pedestrian collisions. The collision scenario was the pedestrian in the standing posture struck by the vehicle at the normal impact height at 40 km/h. Both the cross-sectional force and maximum local strain of the ligaments were changed (Figure 7). The original model had a negative volume problem in the LCL of the contralateral leg (i.e., the leg farther from the vehicle), causing early termination of the simulation. The new model with increased bulk modulus had higher numerical stability. Moreover, the new model had a higher cross-sectional force on the MCL of the ipsilateral leg (i.e., the leg closer to the vehicle) and lower ligament forces on the ACL and LCL of the contralateral leg. The maximum local strains of the ipsilateral MCL of the new and original models were 29% and 35%, respectively (Figure 7A). And the maximum local strain of the contralateral ACL was 39% for the new model and 44% for the original model (Figure 7B). If the ligament strain is used as the failure criterion, the original model tends to overestimate the risk of ligament injury compared to the new model.

### 3.2 Influence of walking in knee bending cases

**FIGURE 8:**
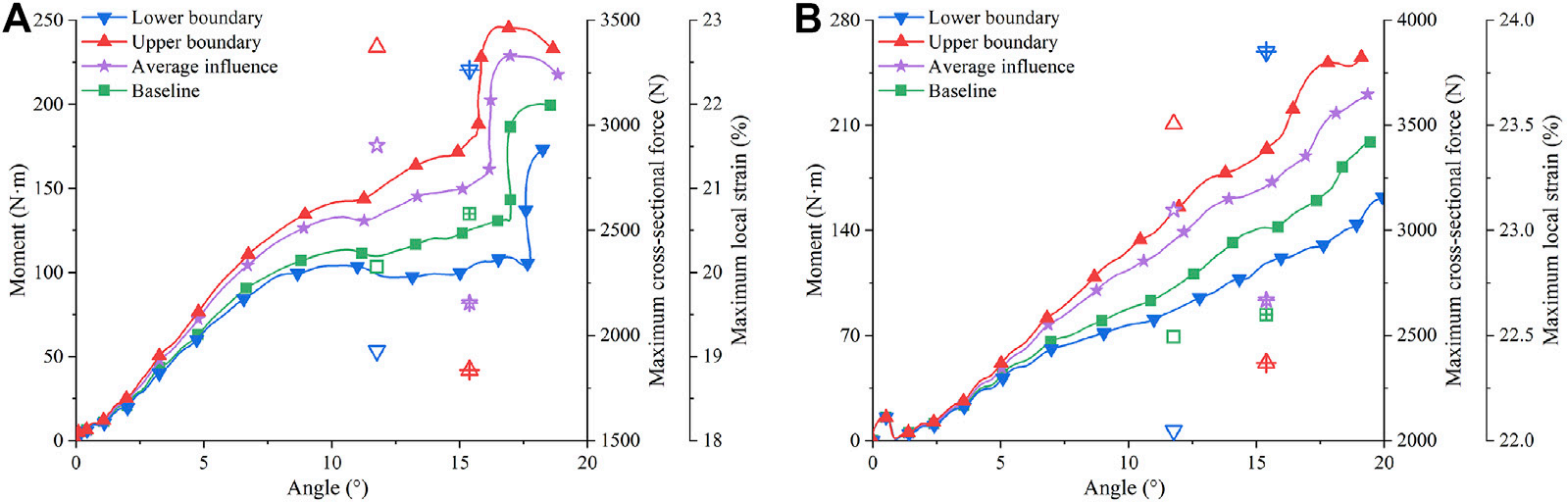
Influence of walking on MCL in bending cases: **(A)** Three-point bending; **(B)** Four-point bending. The hollow markers correspond to the maximum cross-sectional force of the right axis. The markers with + correspond to the maximum local strain of the right axis.

Pedestrian walking affects the knee ligament material properties. Using the material curves determined in Section 2.3, the influence of walking-induced material changes in 3-point and 4-point bending cases is shown in Figure 8. Comparing the simulation results using the “average influence” curves with those using the baseline curves, walking resulted in a 25% and 24% difference in maximum cross-sectional force and a 5% and 0.3% difference in maximum local strain for 3-point bending and 4-point bending cases, respectively. Considering material uncertainty, the simulation results using the upper boundary curves were compared with those using the lower boundary curves. The differences in maximum cross-sectional force increased to 75% for 3-point bending and 71% for 4-point bending, and the differences in maximum local strain increased to 16% for 3-point bending and 6% for 4-point bending. The large discrepancy between the simulation results of the two scaling methods indicates that there is a large uncertainty space in the simulations that use average material properties.

### 3.3 Influence of walking in pedestrian accidents

**FIGURE 9:**
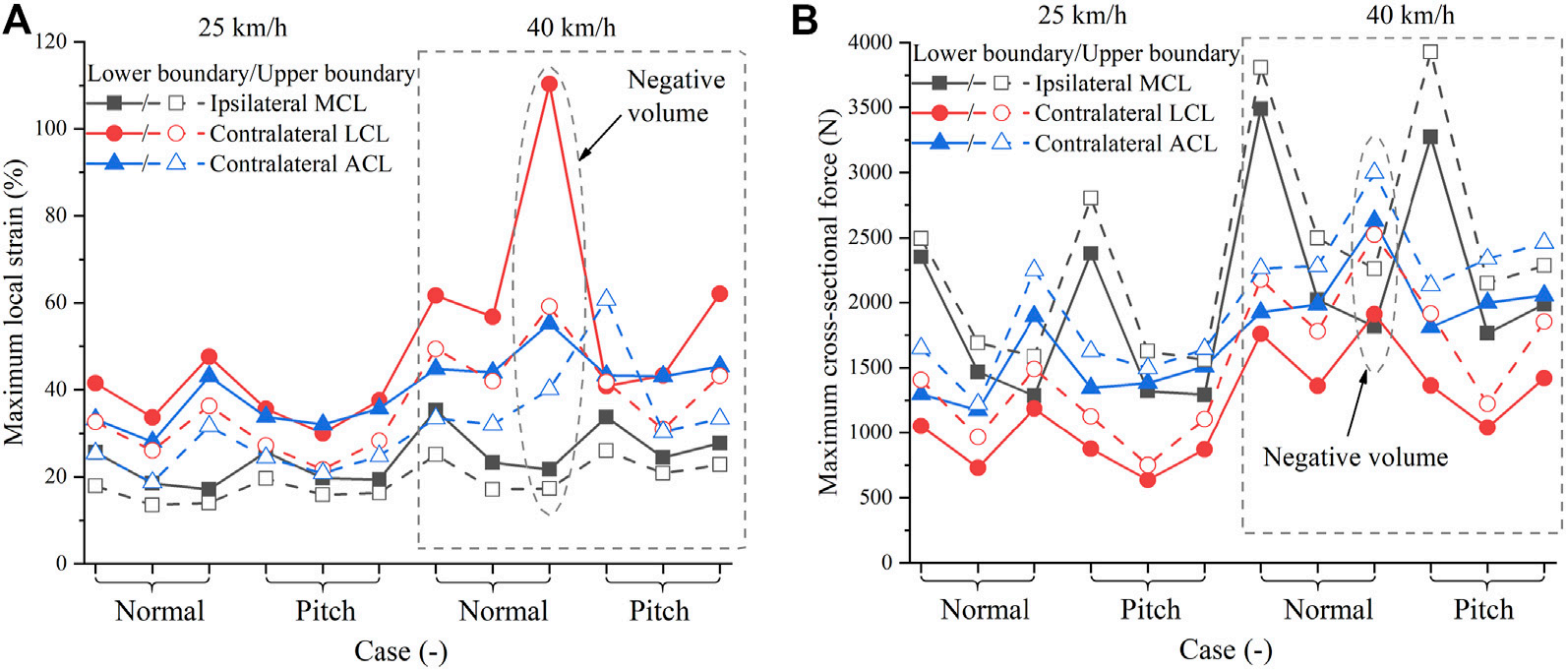
Ligament response in pedestrian accidents: **(A)** Maximum local strain; **(B)** Maximum cross-sectional force. On the horizontal axis, the three notches in each bracket stand for standing, walking (left), and walking (right) postures, respectively, from left to right. The dashed ellipse indicates the case with the negative volume problem.

In car-to-pedestrian collisions, we mainly compared the responses of ipsilateral MCL and contralateral ACL and LCL, because they were the main load-bearing components in pedestrian accidents and, thus, at a greater risk of failure. To find the worst-case scenario, we assessed the difference in ligament responses between the cases using the upper boundary and lower boundary. It is considered as the largest difference that could be attributed to the walking-induced material changes. The maximum local strain and maximum cross-sectional force are plotted in Figure 9. The simulation scenarios included 25 km/h and 40 km/h collision speeds, normal and pitch contact heights, and pedestrian postures of standing, walking (left), and walking (right). Ignoring the cases with the negative volume problem, as the impact speed decreases, both the maximum local strain and maximum cross-sectional force decrease; hence, the injury risk becomes smaller. Comparatively, the injury risk at pitch height is slightly smaller compared to the same case at normal height. So, the introduction of AEBs is beneficial in reducing knee ligament injury risk.

**FIGURE 10:**
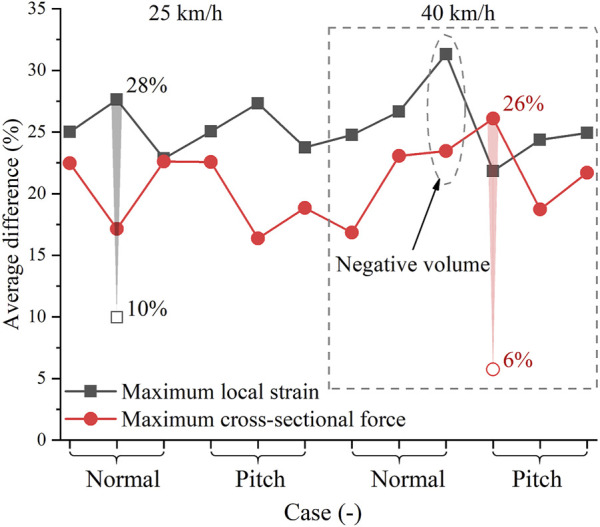
Influence of walking on ligament response in pedestrian accidents. On the horizontal axis, the three notches in each bracket stand for standing, walking (left), and walking (right) postures, respectively, from left to right.

The difference in ligament response for each case was calculated by averaging the absolute differences between the three ligaments. For example, the difference in maximum local strain for each case $∆\varepsilon_{\max,i}$ was calculated as:

$$∆\varepsilon_{\max,i}=\frac{1}{3}\sum_{k=1}^{3}\left|\frac{{\varepsilon \prime }_{\max,i}^{k}-\varepsilon_{\max,i}^{k}}{\varepsilon_{\max,i}^{k}}\right|$$ (2)

where *i* denotes the simulation scenario, corresponding to the horizontal axis in Figure 10. The three ligaments (i.e., ipsilateral MCL and contralateral ACL and LCL) are indicated by *k.* $\varepsilon_{\max,i}^{k}$ is the maximum local strain of *ligament k* at *case i* using lower boundary curve, while ${\varepsilon \prime }_{\max,i}^{k}$ is the maximum local strain of *ligament k* at *case i* using upper boundary curve. The average difference in maximum cross-sectional force was calculated by replacing the maximum local strain with maximum cross-sectional force. The differences in the response for each case are plotted in Figure 10.

In most cases, walking-induced material changes had a greater impact on the maximum local strain than on the maximum cross-sectional force. The changes in the material data from the lower boundary to the upper boundary caused a difference of 22%–28% (mean: 25%) in the maximum local strain and 16%–26% (mean: 21%) in the maximum cross-sectional force. To evaluate the material uncertainty, we singled out the case that was influenced most by walking, and then re-simulated these cases using the average scaling method. The case with the greatest local strain difference was the pedestrian in walking (left) posture hit at 25 km/h at the normal height. Re-simulating this case using the baseline and average influence curves, the maximum local strain difference was reduced from 28% to 10%. The case with the largest cross-sectional force difference was the standing pedestrian impacted at 40 km/h at the pitch height. The maximum cross-sectional difference was reduced from 26% to 6% after using the average scaling method. The average scaling method represents the influence of walking in an average sense, while the upper and lower boundaries somehow represent the maximum possible influence of walking. Walking-induced material changes can lead to at least a 6% deviation in pedestrian ligament response, so it is important to consider walking-induced material changes in the analysis of pedestrian ligament injuries. Most current HBMs or legforms do not consider material changes caused by walking, which is noteworthy when they are used to assess protection effectiveness. Moreover, the influence of walking using the boundary material data was 3–5 times greater than that using the average material data. These results indicate a large uncertainty when using average material curves to analyze pedestrian ligament response (Laz and Browne, 2010). Injury analysis requires consideration of the worst-case scenario, so accounting for material uncertainty in injury analysis is critical to improving the reliability of safety assessments and protection strategies (Davis and Cheong, 2019; Pan et al., 2022).

From material experiments to car-to-pedestrian collisions, the influence of walking decreased. Under the influence of walking, the elastic modulus of the upper boundary was 106% greater than that of the lower boundary (Figure 4A, the modulus of the upper and lower boundary was 377.9 MPa and 183.4 MPa, respectively). Comparatively, in pedestrian collisions, the influence of walking decreased to only about 16%–26% in maximum cross-sectional force and 22%–28% in maximum local strain. The car-to-pedestrian collisions are system-level responses, and so, in addition to the ligament material changes, their overall results are also affected by many other factors in the system. The effect of walking decayed in pedestrian collisions is a good news for pedestrian protection. However, due to the large experimental dispersion of biomaterials, the influence of walking in pedestrian collisions can still be as large as 16%–28%.

## 4 Discussion

We increased the bulk modulus of ligaments in the material card to keep a constant volume in stretching; hence, the numerical stability was improved (Figure 7). However, increasing bulk modulus cannot completely solve the negative volume problem. In pedestrian collision simulations, the ipsilateral MCL and contralateral ACL and LCL are mainly subjected to tensile forces. Among them, the contralateral LCL is most likely to have a negative volume problem. In our simulations, we did not control the hourglass, except in one case where the calculation was terminated due to negative volume (Figure 10). After adjusting the hourglass, the animation looked normal, but the hourglass energy ratio reached 7% of the internal energy. The contralateral LCL had an abnormal maximum local strain of 110.3% when using the lower boundary (Figure 9). Therefore, this case was excluded from the subsequent analysis. Moreover, the increased bulk modulus reduced the accuracy of the compression behavior of the ligaments. In the 3-point and 4-point bending cases, the LCL was mainly in compression. Using the new model, the LCL had a maximum local strain of around 20%, while it was about 6% when using the original model. In reality, ligaments can only withstand tensile forces not much compression. However, this behavior is difficult to be characterized with solid element modeling (Galbusera et al., 2014), which is currently used by THUMS. Neither the original model nor the new model could reliably simulate the compression behavior of knee ligaments. As a compromise, we considered it acceptable to “sacrifice” the accuracy of the compression characterization for tension stability.

In the knee bending cases, the material changes due to walking produced a much greater influence on the maximum cross-sectional force than on the maximum local strain (Figure 8). Comparatively, in pedestrian collisions, walking had a slightly greater impact on the maximum local strain than on the maximum cross-sectional force (Figure 10). This may be caused by the different boundary conditions in knee bending tests and pedestrian collision accidents. In the 3-point and 4-point bending tests, the boundary conditions were controlled by the time-velocity curve; while in the pedestrian collisions, the boundary conditions were mainly controlled by the initial velocity. The former was a stronger constraint than the latter, so the kinematic response of the knee joint in the bending cases was mainly controlled by the boundary condition and less influenced by the ligament material. The small difference in kinematic response caused the small difference in maximum local strain, which together with the large difference in material curves resulted in the large difference in maximum cross-sectional force. In contrast, the lower extremity was less constrained and moved freely in pedestrian collisions. Hence, the difference in maximum local strain in pedestrian collisions was greater. We tried initial velocity to control the boundary condition in the 3-point bending. Without careful recalibration, the maximum force difference was reduced to 36% and the maximum local strain difference increased to 22%. This would worsen the calibration though. With the exercise, we confirmed that the boundary conditions in 3-point and 4-point bending cases enlarged the force difference and reduced the strain difference compared to the car-to-pedestrian collisions.

Using the average scaling method, walking had the greatest influence of 10% on the maximum local strain and 6% on the maximum cross-sectional force. For comparison purposes, the influence of strain rate dependence on the knee ligament response of pedestrians was also investigated. The ligament responses using the baseline curve with rate dependence were compared to those without rate dependence in 25 km/h collisions (Figure 5). The influence of strain rate was greatest when the standing pedestrian was hit at the normal height, with a difference of 4% in maximum local strain and 8% in maximum cross-sectional force. Re-simulating this case at 40 km/h, the difference in maximum local strain was reduced to 2% and in maximum cross-sectional force to 1%. The decreasing influence of strain rate dependence with increasing impact speed may be attributed to the saturation phenomenon of ligament materials, which has been observed in many experiments (Pioletti et al., 1998; Pioletti et al., 1999; Crisco et al., 2002; Bonner et al., 2015). As shown in Figure 3A, from 10/s to 130/s, the strain rate increases by about ten folds, while the tensile curves do not exhibit a significant change. The local strain rate of collateral ligaments at a 40 km/h pedestrian impact is about 30–50/s (Van Dommelen et al., 2005b), entering the saturation state. Hence, the strain rate dependence effect was less than 2% at 40 km/h. The influence of walking was almost independent of the impact velocity (Figure 10). Thus, in high-speed crashes, the influence of walking deserves more attention.

**FIGURE 11:**
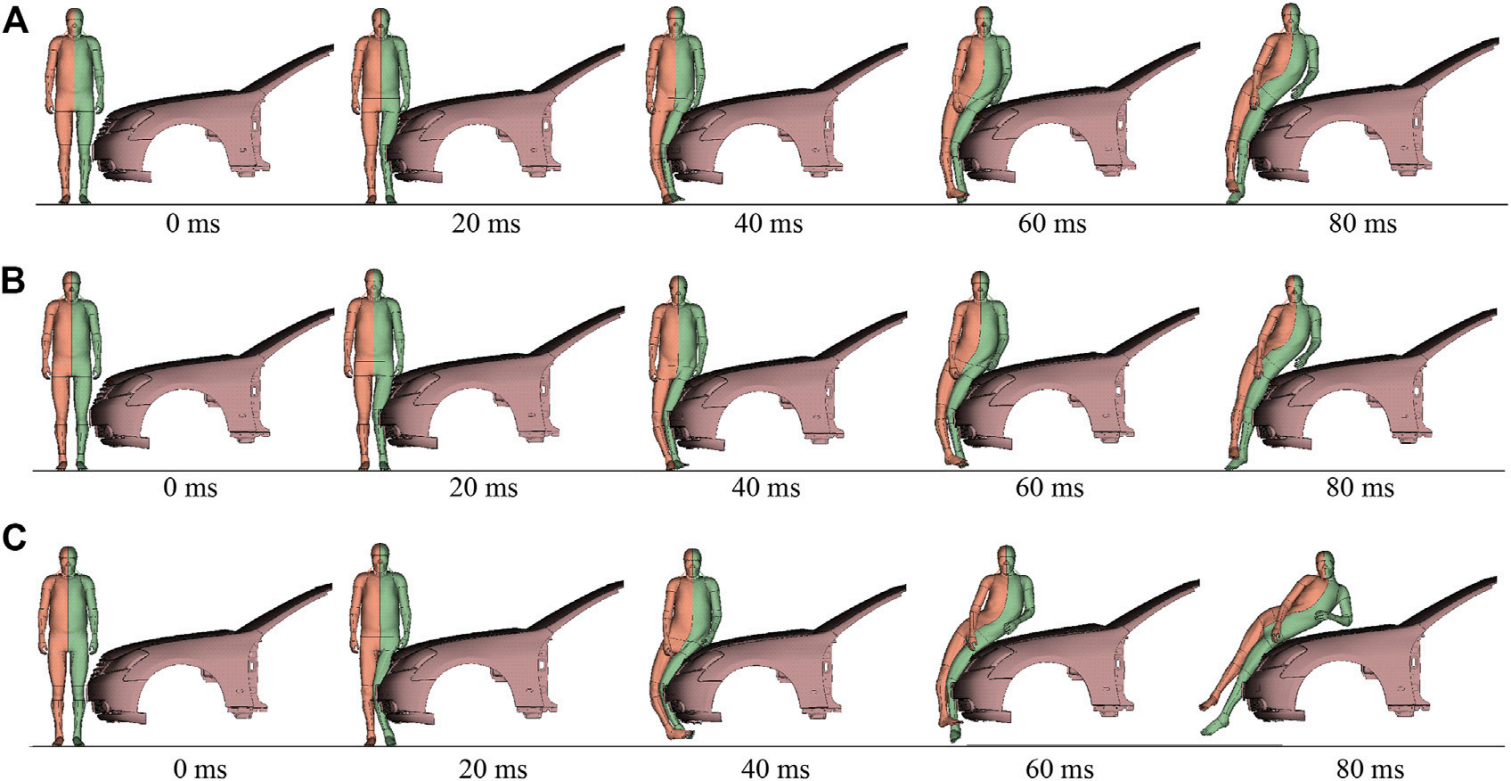
Kinematic response of the standing pedestrian struck by the normal height bumper. **(A)** Upper boundary material curve and 25 km/h; **(B)** Lower boundary material curve and 25 km/h; **(C)** Lower boundary material curve and 40 km/h.

Using the experimental boundary curves, the influence of walking on maximum local strain and maximum cross-sectional force was 22%–28% (mean: 25%) and 16%–26% (mean: 21%), respectively, comparable to the effect of AEB-induced velocity uncertainty on pedestrian ligament response. AEBs mainly decrease ligament injury risk by reducing velocity (Figure 9), which can reduce a 40 km/h collision to 25 km/h. The actual impact can occur at any time during braking, so the impact velocity may be between 25 and 40 km/h. Regarding 25 and 40 km/h as the lower and upper limits of impact velocity, respectively, and reorganizing the results in Figure 9, the maximum local strain difference was 19%–40% (mean: 29%) and the maximum cross-sectional force difference was 30%–42% (mean: 34%) for the ligament response at 25 km/h compared to the same case at 40 km/h. Impact velocity had a more pronounced effect on pedestrian kinematics than material properties (Figure 11). However, the uncertainty of the two factors had a comparable effect on the knee ligament response of pedestrians.

This study has a few limitations. First, we assumed that the scaling factors of the four knee ligaments were the same, as well as the scaling factors for the global and local strain rates of the ligaments. The accuracy and reliability of these assumptions need to be further investigated. Second, failure strains were used in the 3-point and 4-point bending simulations and scaling range decisions, but they were not included in pedestrian collision simulations. Failure strain is also influenced by walking and loading rate (Figure 3A; Figure 4A). The results would be different if failure was included. However, how to consider strain rate dependent failure in simulations deserves further investigation. Third, to improve the reliability for safety assessments (e.g., in virtual testing), it is essential to quantify the uncertainty of deterministic simulation results. This study investigated the material uncertainty in the simplest way, using boundary curves in the simulations. The results show that there is a large uncertainty space in the ligament response simulated using the average material curves. A more detailed study considering material distribution is needed to quantify the output distribution for better safety analysis. Fourth, in addition to viscoelastic behavior, ligaments have other complex mechanical behaviors, such as different loading and unloading curves, and bearing tensile but not compressive forces. These complex behaviors are difficult to be characterized in the current THUMS model. Further optimization of the modeling approach and constitutive models to achieve a more stable and realistic ligament behavior in simulations is important.

## 5 Conclusion

Pedestrian walking affects knee ligament material properties, thus influencing the injury risk of knee ligaments. Based on the THUMS model, we investigated the influence of walking-induced material property changes on ligament responses (i.e., cross-sectional force and local strain) in car-to-pedestrian collisions. The material curves of human knee ligaments were used as a baseline, and the walking effect was abstracted from the material curves of porcine knee MCLs by scaling factors. The influence of walking-induced material changes was more evident in maximum local strain than in maximum cross-sectional force. The greatest influence of walking was 10% on the maximum local strain and 6% on the maximum cross-sectional force, which was more pronounced than the influence of strain rate dependence in high-speed impacts. Further considering the uncertainty in ligament material properties owing to the dispersion of biomaterial tests, the influence of walking on maximum local strain and maximum cross-sectional force may be as large as 28% (mean: 25%) and 26% (mean: 21%), respectively, which was comparable to the influence of AEB-induced velocity uncertainty. Therefore, to improve the reliability of safety assessment and injury analysis, it is important to consider the walking-induced changes in ligament material properties.

## Data Availability

The raw data supporting the conclusion of this article will be made available by the authors, without undue reservation.
